# Differential Cytotoxic Effects of Cell-Free Supernatants of Emerging Pathogens *Escherichia albertii* and *Escherichia fergusonii* on Four Cell Lines Reveal Vero Cells as a Putative Candidate for Cytotoxicity Analysis

**DOI:** 10.3390/microorganisms12112370

**Published:** 2024-11-20

**Authors:** Kandhan Srinivas, Sandeep Ghatak, Kekungu-u Puro, Zakir Hussain, Mosuri Chendu Bharat Prasad, Arockiasamy Arun Prince Milton, Careen Liza Pakyntein, Dadimi Bhargavi, Samir Das, Madesh Angappan, Vanita Lyngdoh, Sabia Khan, Nur Abdul Kader, Umjerksiar Ramshon

**Affiliations:** 1 Division of Veterinary Public Health, ICAR—Indian Veterinary Research Institute, Bareilly 243122, India; jsrinivaskandan@gmail.com (K.S.); bhargavireddy195@gmail.com (D.B.); angappandr@gmail.com (M.A.); 2 Division of Animal and Fisheries Sciences, ICAR Research Complex for NEH Region, Umiam 793103, India; akulepuro@gmail.com (K.-u.P.); zakirh8131@gmail.com (Z.H.); chendumosuri1994@gmail.com (M.C.B.P.); careenp4@gmail.com (C.L.P.); drsamirvph@gmail.com (S.D.); vanitalyngdoh@gmail.com (V.L.); khansabia165@gmail.com (S.K.); nurabdulvets11@gmail.com (N.A.K.); umjer2024@gmail.com (U.R.)

**Keywords:** *Escherichia albertii*, *Escherichia fergusonii*, cytotoxicity, Vero cells, HeLa cells, CDT

## Abstract

*Escherichia albertii* and *Escherichia fergusonii* are recognized as emerging pathogens with zoonotic potential. Despite their increasing importance, there is a paucity of data on the cytotoxicity of these two pathogens. Therefore, in the present study, we investigated the cytotoxic potentials of the cell-free supernatants from 10 *E. albertii* and 15 *E. fergusonii* isolates for their cytotoxic effects on four different cell lines (CHO, Vero, HeLa, and MDCK). All *E. albertii* isolates (100%) and all but one *E. fergusonii* (93.33%) were cytotoxic. *E. albertii* isolates produced similar cytotoxicity titres across the cell lines, whereas the Vero cell was found to be the most sensitive to toxins produced by *E. fergusonii* (*p* < 0.05), followed by HeLa and CHO cells. MDCK was the least sensitive cell line to *E. fergusonii* toxins (*p* < 0.05). PCR detection of cytotoxicity-associated genes (*cdtB*, *stx1,* and *stx2*) indicated uniform possession of *cdtB* gene by all *E. albertii* isolates, while *stx1* and *stx2* genes were harboured neither by *E. albertii*, nor *E. fergusonii*. Taken together, our results provided experimental evidence of the cytotoxic effects of these two emerging pathogens, and Vero cells were identified as an optimal candidate to study the cytotoxic effects of *E. albertii* and *E. fergusonii*.

## 1. Introduction

The genus *Escherichia* comprises 5 species in addition to the well-known and well-studied type species, *Escherichia coli* [1]. Among these, two species—*E. albertii* and *E. fergusonii*—have gained increasing attention as emerging pathogens in recent years [2,3,4]. *E. albertii* is known to cause watery to bloody diarrhoea in humans and is closely associated with epidemic mortality in finches which poses a zoonotic concern [5]. Identification of *E. albertii* from all seven continents hints at the probable ubiquitous nature similar to *E. coli* [3,6,7]. Despite the reports of food-borne disease outbreaks caused by *E. albertii* being geographically limited to Japan until now. The available literature suggest an increase in the geographical range of countries reporting its isolation [8,9,10,11,12]. Moreover, potential misclassifications and misidentifications have paved the way for possible underreporting of the actual burden of *E. albertii* infections [13]. On the other hand, *E. fergusonii* though initially regarded as an opportunistic pathogen has been reported to cause Haemolytic Uraemic Syndrome (HUS)-like conditions in humans in addition to causing post-operative site infections [14]. In animals, opportunistic infections by *E. fergusonii* have also been reported worldwide from various animal sources such as goats, ostriches, pangolins, horses, and cattle [14,15]. Additionally, previous studies have also implicated these two organisms in urinary tract infections in humans [16,17,18].

In addition to their emerging presence in apparently healthy or diseased animals as well as humans, their increasing presence in foods of animal origin has pronounced them as a potential food safety hazard [5,14]. *E. albertii* has been identified from duck meat, chicken, mutton, pork, milk, cheese, oysters, and vegetables [19,20,21,22,23]. On the other hand, reports of isolation of *E. fergusonii*, especially drug-resistant strains from foods of animal origins have been on the rise [14,24,25,26].

Many scientists have attempted to understand the pathogenicity, virulence, and epidemiology of these pathogens through various methods ranging from conventional wet-lab studies to comparative genomics [9,14,27,28]. However, due to the paucity of the available literature on these organisms, researchers frequently resorted to comparisons with *E. coli* in terms of their virulence, as evident from previous reports [29,30]. However, over the years, the virulence potential, mechanism of pathogenesis, and the ability of the organisms to harbour toxin-related genes have become apparent. *E. albertii* harbours intimin encoded by *eae* gene, a tripartite cytolethal distending toxin (*cdtA*, *cdtB* and *cdtC*), shiga-toxins (*stx2a* and *stx2f*), and Enteroaggregative *E. coli* heat-stable toxin 1 (EAST1) encoded by *astA* [5,29,31]. *E. fergusonii,* on the other hand, was found to harbour heat-stable toxin (STa) and heat-labile toxin (LT) [30,32,33]. Despite their emerging importance, only a few studies have ventured into the in vitro virulence properties of cell culture systems [29,34,35].

Therefore, the aim of this study was to compare the cytotoxic effects of *E. albertii* and *E. fergusonii* on four commonly used cell lines and to identify the most suitable cell line for phenotypically studying the cytotoxicity of these two organisms.

## 2. Materials and Methods

### 2.1. Strains and Controls

A total of 10 *E. albertii* and 15 *E. fergusonii* strains recovered from diverse sources were used in this study (Table 1). The isolation of *E. albertii* and *E. fergusonii* were undertaken as per previously published methods [28,36,37]. Briefly, bacteriological isolation and identification of *E. albertii* involved enrichment of the sample in novobiocin-modified EC broth (nmEC) at 42 °C for 24 h followed by plating on Xylose-Rhamnose-Melibiose (XRM) MacConkey agar. Similarly, the enrichment for *E. fergusonii* was conducted in Luria broth at 37 °C for 24 h followed by plating on Simmons citrate agar supplemented with 2% adonitol. Plates were incubated at 37 °C for 48 h for both *E. albertii* and *E. fergusonii.* The presumptive colony characteristics on respective media were pale colourless colonies for *E. albertii* and yellowish orange colonies for *E. fergusonii.* Additionally, biochemical tests such as ornithine decarboxylase, lysine decarboxylase, cellobiose, and arabinose fermentation tests were also used to phenotypically confirm the identification of the isolates. PCR-based confirmation was subsequently undertaken by targeting the 393 bp DNA-binding transcriptional activator of the cysteine biosynthesis gene for *E. albertii* and a 575 bp palmitoleoyl-acyl carrier protein for *E. fergusonii* [9]. Known cytotoxic STEC isolates (G97 and G109) from our previous study [38], harbouring both *stx1* and *stx2* genes were taken as a positive control for this study. *E. coli* DH5α (Thermo Fisher Scientific, Waltham, MA, USA) served as the negative control throughout the study. Positive and negative control strains were tested on the Vero cells for the presence and absence of cytotoxic effects, respectively.

### 2.2. Cell Lines

Four different cell lines, Vero (RRID: CVCL_0059), CHO (RRID: CVCL_0213), HeLa (RRID: CVCL_0030), and MDCK (RRID: CVCL_0422), were procured from the National Centre for Cell Sciences, Pune, India. These four cell lines were chosen because a number of available reports indicated their previously documented use in studying the cytotoxicity of various bacterial pathogens and their availability in laboratories [39,40,41,42,43,44,45,46,47,48,49,50,51,52,53,54].

### 2.3. Preparation of Cell-Free Supernatant

Cell-free supernatants (CFS) of the isolates were prepared as described by Ghatak et al. [55] with suitable modifications. The isolates were inoculated on Tryptone Soy Broth (HiMedia, Mumbai, India) and were grown at 37 °C for 14–16 h. An aliquot of 6 mL broth was centrifuged at 10,000× *g* for 20 min at 4 °C. The supernatants were transferred to fresh sterile tubes and were filtered through a syringe filter (0.22 µm) (Corning Inc., New York, NY, USA). A sterility check of the CFS was performed by inoculating in sterile Luria Broth (HiMedia, India) and incubated at 37 °C overnight. The pH of the CFSs was also checked after preparation, before storage at −20 °C, and before application to ensure that the pH remained within 7.2 to 7.4. Additionally, the CFS preparations were also subjected to SDS-PAGE (10%) analysis followed by Periodic acid Schiff staining to check for the presence of LPS [56].

### 2.4. Assessment of Cytotoxicity

The cytotoxic potentials of *Escherichia albertii* and *Escherichia fergusonii* isolates were tested on Chinese Hamster Ovary (CHO), African green monkey kidney (Vero), Human cervical carcinoma (HeLa), and Madin Darby Canine Kidney (MDCK) cell lines following the methodology reported previously [55] with necessary modifications. The cell lines were propagated in Eagle’s minimum essential medium (EMEM) (Merck-Sigma, St. Louis, MO, USA) with 10% and 2% foetal bovine serum (FBS) (Merck-Sigma, USA) during growth and maintenance, respectively. Cytotoxic potentials were assessed on 96-well tissue culture plates (Corning Inc., USA). Cells were inoculated in individual wells so as to achieve 90 percent confluency. The CFSs were treated with polymyxin (30 µg/mL for 12 h) according to the method described by Lu et al. [57] before adding it to the cells to eliminate the possibility of residual Lipopolysaccharides in the CFS preparations. Thereafter, serial double- fold dilutions of the CFS were prepared in a 96-chamber deep well plate (Corning Inc., USA) using EMEM with 2% FBS as diluent. FBS was added to prevent any possible residual effect of trypsin in case it was present in the bacteriological media used for CFS preparation. The media used for culturing the cells in the 96 well plates were discarded, and 100 µL of CFS dilutions were added to each well in duplicate. The STEC isolates (G97 and G109), treated the same way as the other isolates, were used as the positive controls, while *E. coli* DH5α was used as the negative control. Uninoculated sterile Tryptone Soy Broth (HiMedia, India) was used as the broth control, and EMEM with 2% FBS was added to the wells designated as cell control. The plates were incubated at 37 °C in a 5% CO_2_ atmosphere in a CO_2_ incubator (Galaxy, New Brunswick, NJ, USA), and the readings were taken at 6 h intervals in an inverted microscope (Zeiss, Jena, Germany) for up to 72 h for any deviations in the cellular morphology (cytopathic effect). CFS preparations that induced cytopathic effects in at least 50% of cells up to a dilution of 1:8 or higher were recorded as positive and further titrated if necessary. The cytotoxicity titre of each individual CFS preparation was determined as the reciprocal of the highest dilution that induced cytopathic effects in at least 50% of cells. The titre values were then log-transformed to the base 2 followed by the addition of unity. Additionally, a Crystal Violet assay was performed at the end of the experiment to evaluate the viability of the cells post-treatment [58].

### 2.5. Detection of Cytotoxicity Associated Toxin Genes

*E. albertii* and *E. fergusonii* isolates used in the current study were investigated for the presence of three known toxin genes *cdtB*, *stx1*, and *stx2* as described previously [59,60]. Briefly, DNA from bacterial cells grown overnight at 37 °C in Tryptone Soy Broth (HiMedia, India) were extracted using PureLink™ Genomic DNA Mini Kit (Thermo Fisher Scientific, USA). PCR for *cdtB* gene was performed employing primers F: 5′-GAAAGTAAATGGAATATAAATGTCCG-3′ and R: 5′-AAATCACCAAGAATCATCCAGTTA-3′ with annealing temperature of 54 °C [59]. A known *cdtB* gene-positive *E. albertii* isolate (EA21) available in our laboratory was used as a positive control, while sterile nuclease-free water was used as a negative control. Similarly, PCRs for *stx1* and *stx2* genes were performed using primers stx1F: 5′-CAGTTAATGTGGTGGCGAAGG-3′, stx1R: 5′-CACCAGACAATGTAACCGCTG-3′ and stx2F: 5′-ATCCTATTCCCGGGAGTTTACG-3′, stx2R: 5′-GCGTCATCGTATACACAGGAGC-3′ with annealing at 60 °C [60]. DNA from *E. coli* isolates (G97) and sterile nuclease-free water were used as positive and negative controls, respectively. PCR products were analysed by electrophoresing in 1.5% agarose gel and were visualized under UV illumination (DNR Mini Lumi, Jerusalem, Israel). 

### 2.6. Statistical Analysis

Comparison of means was undertaken with the help of Analysis of Variance (ANOVA). Fisher’s least significant difference (LSD) test was used as the post-hoc test to ascertain the groups with statistically significant differences (*p* < 0.05). The statistical analysis was undertaken in R software version 4.2.2 (https://cran.r-project.org/) accessed on 1 March 2024.

## 3. Results

### 3.1. Phenotypic and Genotypic Confirmation of E. albertii and E. fergusonii

*E. albertii* and *E. fergusonii* isolates used in this study were isolated from various sources (chicken intestine, chicken cloacal, duck faecal, chicken meat) and were identified based on colony characteristics on specific media such as pale colourless colonies of *E. albertii* on XRM-MacConkey agar and yellowish orange colonies on SCA agar. Additionally, biochemical tests also supported the phenotypic identification. Further confirmation was based on conventional PCR where amplicon sizes of 393 bp and 575 bp were observed for *E. albertii* and *E. fergusonii*, respectively (Supplementary Figure S1).

### 3.2. Cytotoxicity of E. albertii and E. fergusonii on CHO, Vero, HeLa, and MDCK Cells

**Figure 1:**
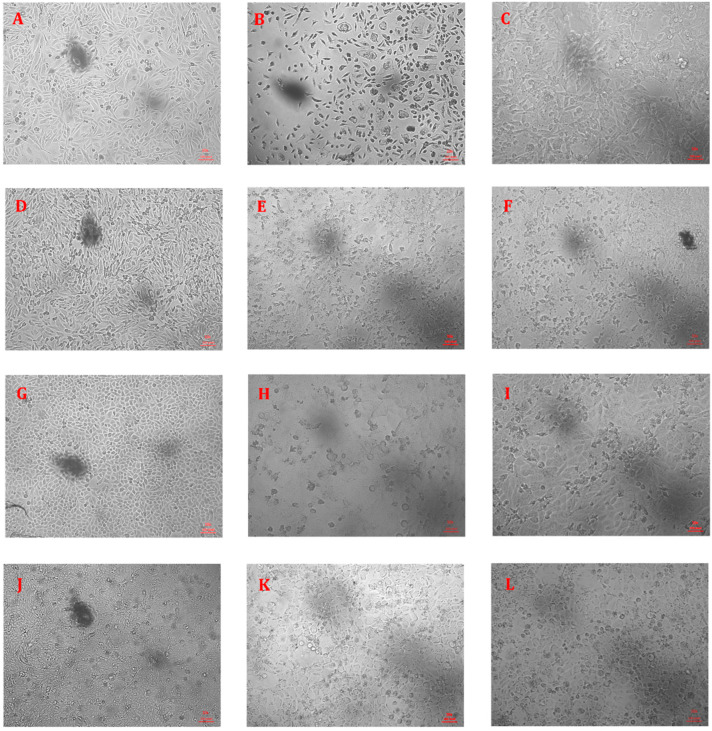
Inverted microscope images of healthy control cells and infected cells showing cytopathic effects. (**A**) Healthy CHO cells (cell control); (**B**) CHO cells treated with CFS of isolate EA1 showing cell lysis and ballooning; (**C**) CHO cells treated with CFS of isolate EF4 showing cell coalescence and rounding; (**D**) Healthy Vero cells (cell control); (**E**) Vero cells treated with CFS of isolate EA1 showing ballooning of cells and plaque formation; (**F**) Vero cells treated with CFS of isolate EF4 showing rounding of cells and increased nucleation; (**G**) Healthy HeLa cells (cell control); (**H**) HeLa cells treated with CFS of EA1 showing ballooning and rounding of cells; (**I**) HeLa cells treated with CFS of EF4 showing rounding of cells; (**J**) Healthy MDCK cells; (**K**) MDCK cells treated with CFS of isolate EA1 showing rounding of cells; (**L**) MDCK cells treated with CFS of isolate EF4 showing rounding of cells.

The ability of the cell-free supernatant of *E. albertii* and *E. fergusonii* isolates to induce a cytopathic effect on the cells was studied on four different cell lines. The pH of the CFS used in the study was monitored to remain within 7.2 to 7.4 during the experiment. Microscopic observation of the treated cells revealed cytotoxic effects such as shrinking of the cells, detachment of the cells, condensation of the nucleus, and shriveling (Figure 1). Additionally, the cytotoxic effects were also evaluated by Crystal Violet staining which showed results concordant with the microscopic evaluation (Supplementary Figure S2). SDS-PAGE analysis of CFS preparations followed by PAS staining revealed an absence of LPS in CFS (Supplementary Figure S3). Moreover, to prevent any possible cytotoxic effect of LPS we treated the CFS preparations with polymyxin as previously recommended by Lu et al. [57].

**Figure 2:**
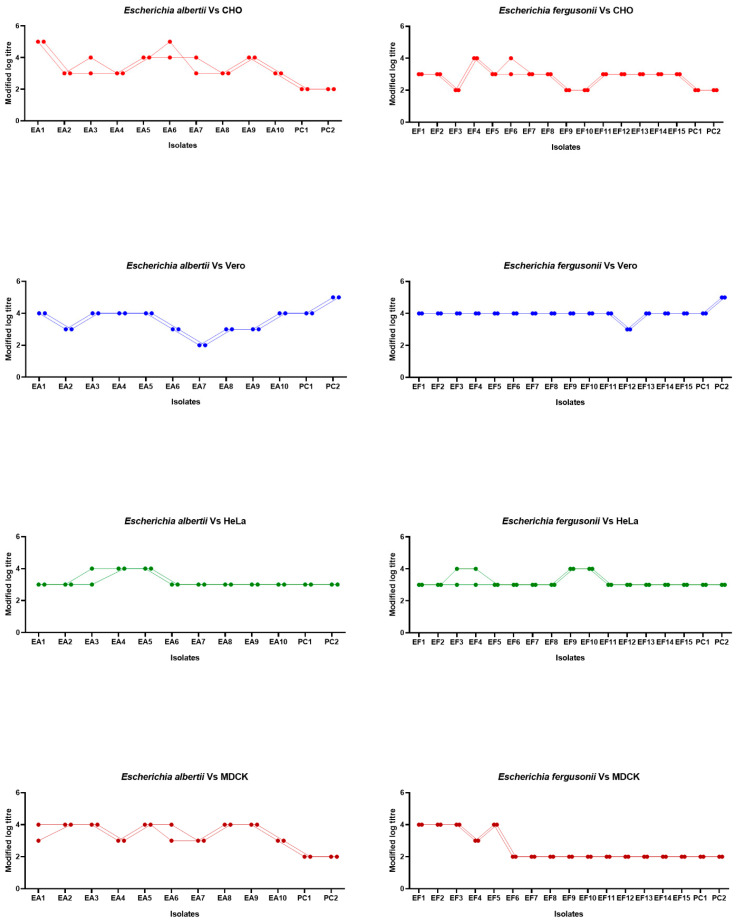
Graphical representation of cytotoxic titres of CFS replicates on different cell lines.

Cytotoxicity was observed in all *E. albertii* isolates (100%) and in all but one *E. fergusonii* isolate (14/15, 93.33%). Overall, Vero cells were found to be most sensitive to the cytotoxic elements of all the isolates (mean = 3.7200 ± 0.10832; *p* < 0.05) followed by HeLa (3.2200 ± 0.07681), CHO cells (3.2000 ± 0.14142) and MDCK (3.000 ± 0.18028). Mean comparison by ANOVA (Table 2) revealed that among *E. albertii* isolates, no significant difference was observed between the cytotoxic sensitivity of cell lines (*p* = 0.409). On the contrary, significant differences were observed among cytotoxicity titres of *E. fergusonii* on various cell lines (*p* < 0.05). LSD test identified that Vero cells (3.9333 ± 0.06667) were the most sensitive to the cytotoxic effects exerted by toxins of *E. fergusonii* followed by HeLa (3.2000 ± 0.09512) and CHO (2.9000 ± 0.13973); whereas, MDCK cell lines were the least sensitive (2.6000 ± 0.23503; *p* < 0.05). ANOVA (Table 3) revealed a statistically significant difference among *E. albertii* and *E. fergusonii* isolates in relation to the positive controls on CHO, Vero, and MDCK cell lines (*p* < 0.05) but not on HeLa cells. On subsequent LSD analysis, *E. albertii* isolates were statistically more virulent on CHO and MDCK cells when compared to *E. fergusonii* isolates as well as the positive controls (*p* < 0.05). On the Vero cell system, *E. fergusonii* isolates were statistically more virulent than *E. alberti* (*p* < 0.05). Interestingly, *E. fergusonii* and the positive controls (STEC isolates) also behaved similarly in the Vero cell system. The HeLa system did not exhibit any statistically significant difference in terms of virulence, indicating that all three groups of isolates behaved the same way (Figure 2).

### 3.3. Occurrence of Toxin Genes Associated with Cytotoxicity in E. albertii and E. fergusonii

PCR was employed to detect molecular signatures of cytotoxicity-associated genes such as *cdtB*, *stx1*, and *stx2* in the isolates. The virulence gene *cdtB* coding for CDT toxin was identified in all *E. albertii* isolates showing an amplicon size of 467 bp (Supplementary Figure S4); however, none of the *E. fergusonii* isolates showed the presence of this gene. Similarly, PCR screening for *stx1* (Supplementary Figure S4) and *stx2* genes (Supplementary Figure S4) yielded positive amplification only in positive control, and none of the isolates harboured *stx1* or *stx2* genes.

## 4. Discussion

In the present study, we evaluated the cytotoxic potential of *E. albertii* and *E. fergusonii* on four cell lines, including two kidney-based cell lines, one ovarian cell line, and one human-origin carcinoma cell line. The choice of these four cell lines was based on the available reports of their use in studying the cytotoxicity of bacterial pathogens [39,40,41,42,43,44,45,46,47,48,49,50,51,52,53,54]. CFS of the broth in which the bacteria were grown were treated with polymyxin B to ensure that the observed cytotoxic effects were indeed due to the cytotoxic factors secreted by the bacterial cells into the broth. Additionally, having the pH of the CFS within the range of 7.2 to 7.4 ensured that the cytotoxicity was not due to the effect of pH.

Analysis of our results revealed cytotoxic effects by the CFS of *E. albertii* isolates on all four cell lines with cytotoxicity titres ranging between 3.25 ± 0.13 (HeLa) and 3.65 ± 0.22 (CHO) indicating no significant difference in cytotoxicity across the cell lines. This uniformity of cytotoxic response might be due to the universal presence of Cytolethal distending toxin (CDT), which forms a constant component of the virulence arsenal of *E. albertii.* In fact, the *cdtB* gene has previously been used as a molecular target for the screening of *E. albertii* [34]. Conventional PCR-based screening of the *E. albertii* strains also confirmed the presence of *cdtB* gene in all *E. albertii* isolates used in the current study. CDT is a type of AB2 toxin that uniquely disrupts the cell cycle and causes distension of the cells [61]. At higher concentrations, the toxin might even cause cell death through the activation of an apoptotic cascade [62]. Few experiments have linked this toxin to tumorigenesis [63]. They are usually produced by Gram-negative bacteria (more than 30 genera of gamma and epsilon bacteria members) such as *Campylobacter jejuni*, *Haemophilus ducreyii*, *Campylobacter coli*, *E. coli*, *Shigella dysenteriae*, *Salmonella enterica*, etc. [62,64,65]. CDT has been found to produce profuse effects on cell lines such as CHO, HeLa, Vero, and MDCK [62,66]. The presence of *stx2* gene is comparatively rare in frequency when compared to the near-omnipresence of *cdtB* gene in *E. albertii* [5], which may also contribute to the absence of significant differences across the cell lines for *E. albertii.* Incidentally, none of the isolates in the present study harboured *stx* genes. In contrast to *E. albertii*, very few studies have focussed on elucidating the virulence of *E. fergusonii* [29,33]. Heat stable (STa) and heat-labile toxins (LT) comprise the major members of the virulence armory of *E. fergusonii* elucidated until the date [30,33]. Our results revealed that Vero cells exhibited significantly higher sensitivity to the cytotoxic effects of *E. fergusonii* (3.93 ± 0.07), followed by HeLa cells, which showed the next highest sensitivity (3.20 ± 0.10). Additionally, no statistically significant difference was noticed between the positive controls, and *E. fergusonii* isolates on Vero cells and HeLa cells. The increased sensitivity of Vero cells and HeLa cells to the CFS of *E. fergusonii* isolates over the other cell lines is a new finding that emanates from the present study that needs future research into the possible mechanisms for such selective cytotoxic effects. Incidentally, during the last 5 years, at least two cases resembling Haemolytic Uraemic syndrome in humans due to *E. fergusonii* were identified in South Korea [18,67]. HUS is a clinical condition usually associated with enterohaemorrhagic *E. coli* harbouring verotoxin genes such as *stx1* and *stx2*. Therefore, the heightened verotoxic nature of the CFS of *E. fergusonii,* despite the absence of *stx1* and *stx2,* was indeed intriguing and possibly indicates the presence of yet-to-be-recognized virulence factors that selectively and aggressively affect the cell lines of primatal origin (Vero and HeLa cells), compared to the cells of cricetine origin (CHO cells) and canine kidney-derived MDCK cells. Differences in cell line receptors based on their origins might perhaps contribute to the variable sensitivity of primate-origin cells compared to cells from other origins. Our study provided important experimental evidence suggesting that *E. fergusonii* may lead to conditions resembling HUS in humans and could pose a serious public health threat. However, further studies are needed to elucidate the underlying mechanisms. We also observed differences among the two organisms (*E. albertii* and *E. fergusonii*) across the cell lines, which were likely due to variations in toxin genes harboured by these organisms. Similarly, the increased cytotoxicity of *E. albertii* on CHO and MDCK, when compared to *E. fergusonii*, could well be due to the presence of *cdtB* gene in *E. albertii*, and its absence in *E. fergusonii*.

## 5. Conclusions

Overall, our study provided experimental evidence of the cytotoxic effects of emerging pathogens, *E. albertii* and *E. fergusonii,* on four different cell lines (CHO, Vero, HeLa, and MDCK). Although all four cell lines showed similar cytotoxicity towards *E. albertii*, significant differences were observed among the cell lines in their response to the cytotoxic effects of *E. fergusonii*, with Vero cells demonstrating the highest sensitivity. Therefore, taken together, Vero cells were identified as a suitable option to study the cytotoxic effects of *E. albertii* and *E. fergusonii* though further research is needed to unravel the underlying mechanisms.

## Figures and Tables

**Table 1:** Source and characteristics of isolates used in the current study.

Isolate	Source	Virulence Genes		
		*cdtB*	*stx1*	*stx2*
EA1	Chicken intestine	+	−	−
EA2	Chicken intestine	+	−	−
EA3	Chicken intestine	+	−	−
EA4	Chicken intestine	+	−	−
EA5	Chicken intestine	+	−	−
EA6	Chicken meat	+	−	−
EA7	Chicken meat	+	−	−
EA8	Chicken meat	+	−	−
EA9	Chicken intestine	+	−	−
EA10	Chicken intestine	+	−	−
EF1	Chicken intestine	−	−	−
EF2	Chicken intestine	−	−	−
EF3	Duck faecal	−	−	−
EF4	Chicken meat	−	−	−
EF5	Chicken meat	−	−	−
EF6	Chicken meat	−	−	−
EF7	Chicken meat	−	−	−
EF8	Chicken meat	−	−	−
EF9	Chicken meat	−	−	−
EF10	Chicken cloacal	−	−	−
EF11	Duck faecal	−	−	−
EF12	Chicken intestine	−	−	−
EF13	Chicken intestine	−	−	−
EF14	Chicken intestine	−	−	−
EF15	Chicken intestine	−	−	−

**Table 2:** Mean comparison of cytotoxicity titres obtained in the current study across different cell lines.

Organism	CHO	Vero	HeLa	MDCK	*p* Value
*E. albertii*	3.65 ± 0.22	3.40 ± 0.22	3.25 ± 0.13	3.60 ± 0.14	0.41
*E. fergusonii*	2.90 ± 0.13 ^b^	3.93 ± 0.07 ^c^	3.20 ± 0.10 ^b^	2.60 ± 0.24 ^a^	0.00 **

** represents statistical significance at *p* < 0.01 level. Mean values with different alphabetical superscripts in the same row represent significant differences among cell lines.

**Table 3:** Mean comparison of cytotoxicity titres obtained in the current study among different organisms.

Organism	CHO	Vero	HeLa	MDCK
*E. albertii*	3.65 ± 0.22 ^b^	3.40 ± 0.22 ^a^	3.25 ± 0.13	3.60 ± 0.14 ^b^
*E. fergusonii*	2.90 ± 0.13 ^a^	3.93 ± 0.07 ^b^	3.20 ± 0.10	2.60 ± 0.24 ^a^
Control	2.00 ± 0.00 ^a^	4.50 ± 0.35 ^b^	3.00 ± 0.00	2.00 ± 0.00 ^a^
*p* value	0.002 **	0.009 **	0.704	0.004 **

** represents statistical significance at *p* < 0.01 level. Mean values with different alphabetical superscripts in the same column represent significant differences among organisms.

## Data Availability

The original contributions presented in the study are included in the article/Supplementary Materials. Further inquiries can be directed to the corresponding authors.

## Supplementary Materials

The following supporting information can be downloaded at: https://www.mdpi.com/article/10.3390/microorganisms12112370/s1, Figure S1A: Molecular detection of DNA-binding transcriptional activator of cysteine biosynthesis gene using PCR. Lane L—100 bp ladder|Lane 1—Known positive control with an amplicon of 393 bp|Lane 2—Negative template control|Lane 3—Isolate EA1; Figure S1B: Molecular detection of palmitoleoyl-acyl carrier protein using PCR. Lane L—100 bp ladder|Lane 1—Known positive control with an amplicon of 575 bp|Lane 2—Negative template control|Lane 3—Isolate EF4; Figure S2: Crystal Violet staining of the cells post-treatment to assess the viability A: CHO cells; B: Vero cells; C: HeLa cells; D: MDCK cells; Figure S3A: Molecular detection of cytolethal distending toxin subunit B (*cdtB*) using PCR. Lane L—100 bp ladder|Lane 1—Known positive control with an amplicon of 467 bp|Lane 2—*E. albertii* isolate showing positive for *cdtB* gene|Lane 3—Negative template control; Figure S3B: Molecular detection of shigatoxin 1 gene (*stx1*) using PCR. Lane L—100 bp ladder|Lane 1—Negative template control|Lane 2—Known positive control with an amplicon of 348 bp|Lanes 3–6—*E. albertii* and *E. fergusonii* isolates showing negative for *stx1* gene; Figure S3C: Molecular detection of shigatoxin 2 gene (*stx2*) using PCR. Lane L—100 bp ladder|Lane 1—Negative template control|Lane 2—Known positive control with an amplicon of 584 bp|Lanes 3–6—*E. albertii* and *E. fergusonii* isolate showing negative for *stx2* gene; Figure S4: SDS-PAGE analysis detecting the presence of LPS. Lane 1: Protein marker (M); Lane 2: Lipopolysaccharide (LPS), Lane 3: EA1 (S1); Lane 4: EA2 (S2); Lane 5: EF1 (S3); Lane 6: EF2 (S4).
