# Supplementary figures and images for: Differential Cytotoxic Effects of Cell-Free Supernatants of Emerging Pathogens Escherichia albertii and Escherichia fergusonii on Four Cell Lines Reveal Vero Cells as a Putative Candidate for Cytotoxicity Analysis
Source: Microorganisms. 2024 Nov 20;12(11):2370. doi: 10.3390/microorganisms12112370 (PMC11596466; doi:10.3390/microorganisms12112370)

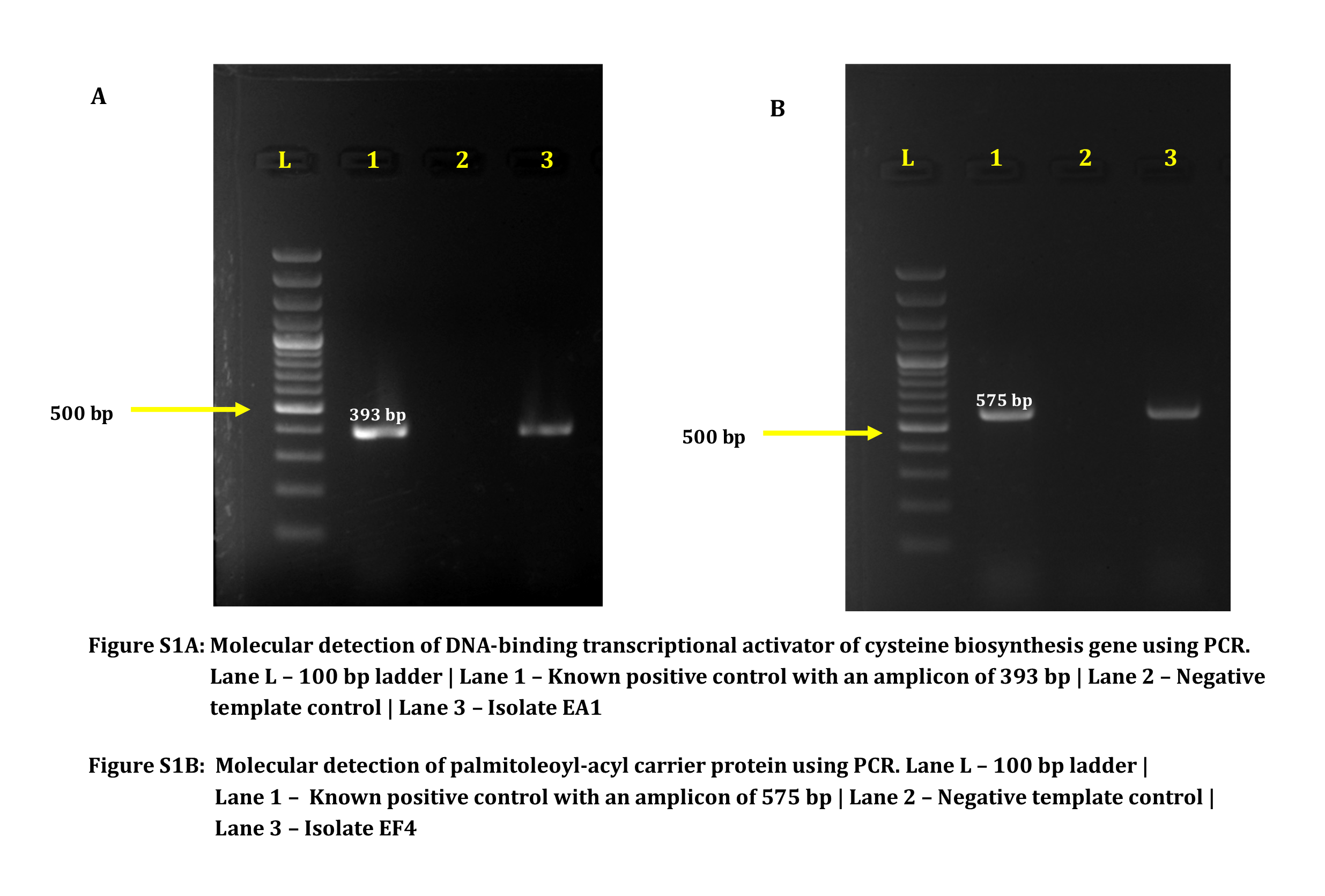

Supplement: Supplementary file 1 [file microorganisms-12-02370-s001.zip › Supplementary Figure S1.tif]

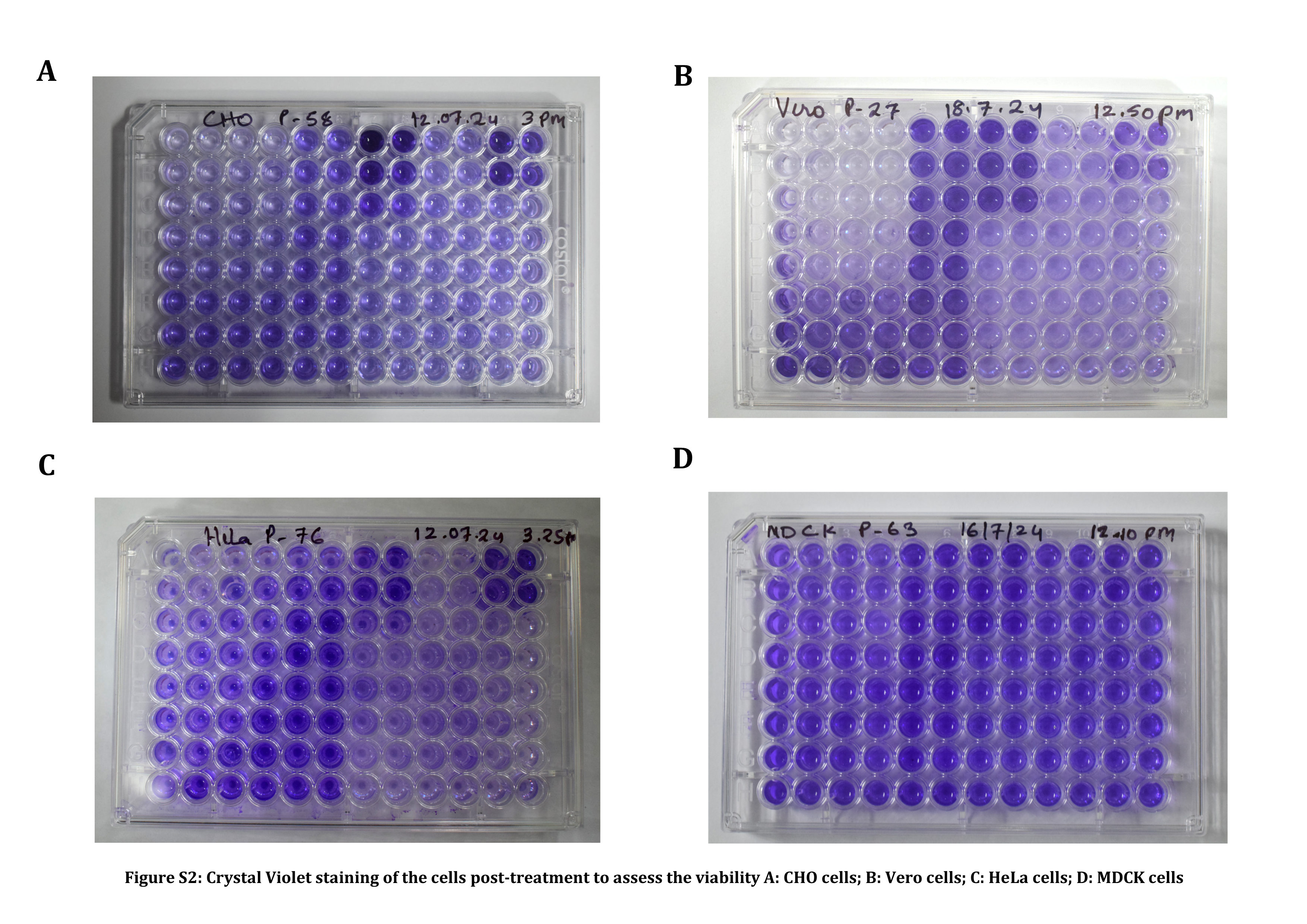

Supplement: Supplementary file 1 [file microorganisms-12-02370-s001.zip › Supplementary Figure S2.tif]

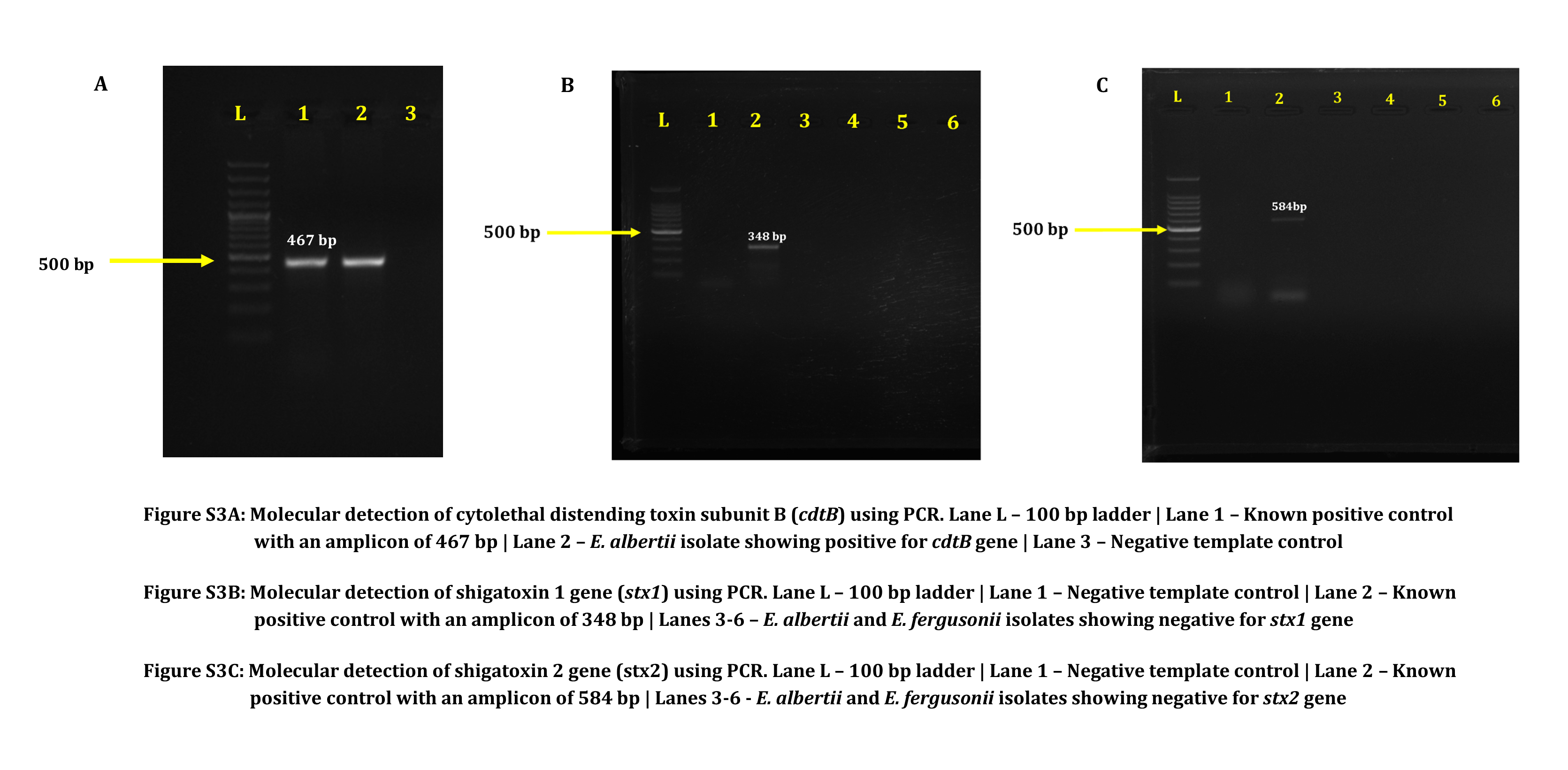

Supplement: Supplementary file 1 [file microorganisms-12-02370-s001.zip › Supplementary Figure S3.tif]

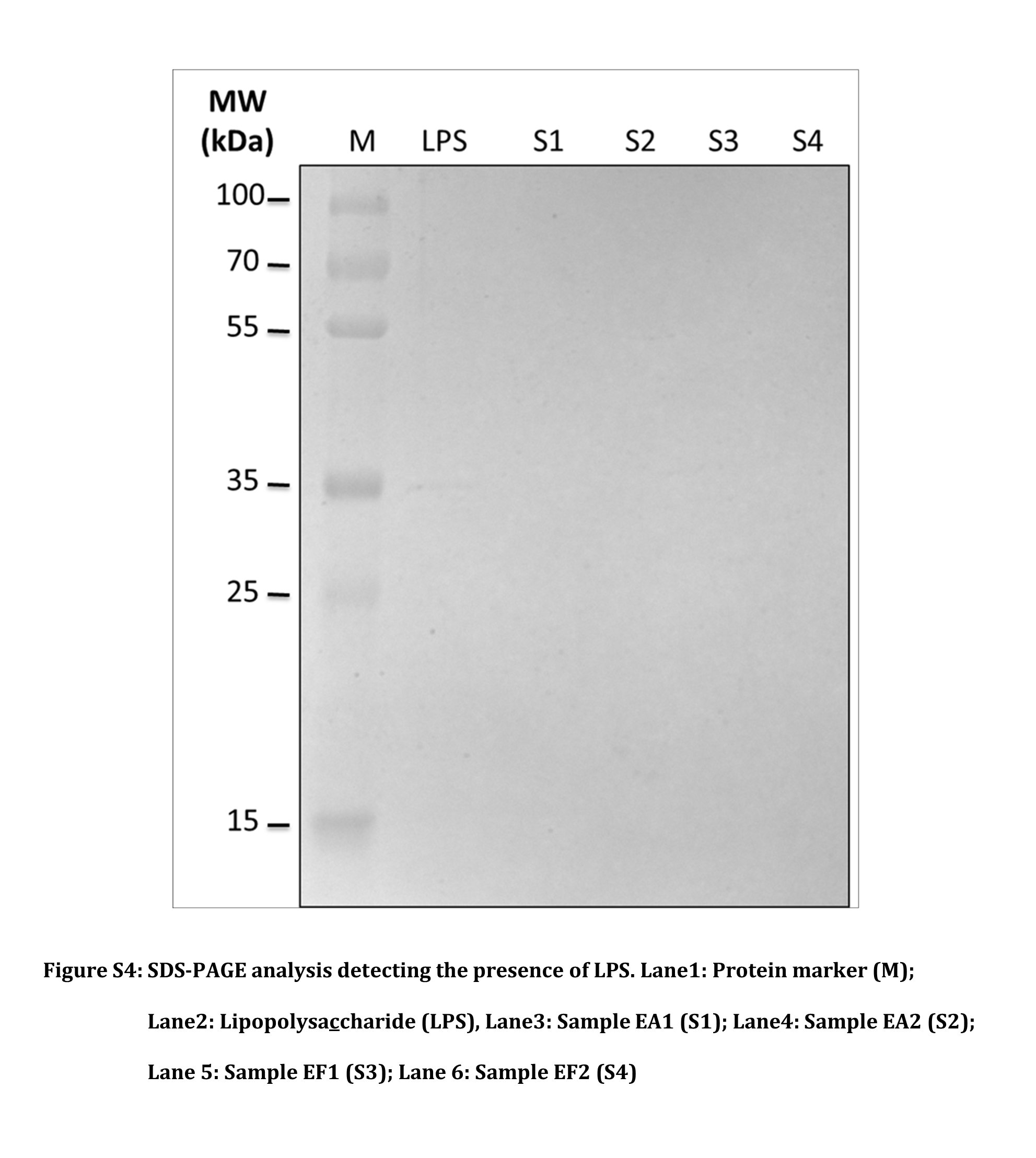

Supplement: Supplementary file 1 [file microorganisms-12-02370-s001.zip › Supplementary Figure S4.tif]
